# The perception of disability by community groups: Stories of local understanding, beliefs and challenges in a rural part of Kenya

**DOI:** 10.1371/journal.pone.0182214

**Published:** 2017-08-03

**Authors:** Karen Bunning, Joseph K. Gona, Charles R. Newton, Sally Hartley

**Affiliations:** 1 School of Health Sciences, University of East Anglia, Norwich, United Kingdom; 2 Centre for Geographic Medicine Research (Coast), Kenya Medical Research Institute, Kilifi, Kenya; 3 Department of Psychiatry, University of Oxford, Oxford, United Kingdom; 4 London School of Hygiene and Tropical Medicine London, United Kingdom; Universita degli Studi di Perugia, ITALY

## Abstract

Cultural narratives on disability have received much attention over the past few decades. In contexts of poverty, limited information and everyday challenges associated with having, or caring for someone with a disability, different understandings have emerged. A project was set up to promote disability awareness in neighborhood communities in a rural part of Kenya, using a process of reflection and education. This paper reports on the first aspect–reflection. The aim was to investigate local understanding of disability as a co-constructed concept. The research questions were: 1. What cultural beliefs shape local understanding of disability? 2. What challenges are perceived to be associated with disability? A phenomenological approach was adopted. Focus group discussions were conducted with twenty-one community groups involving 263 participants and audio-recorded. The data were transcribed and thematic analysis was carried out. Visual maps were created to illustrate any interconnections, before establishing the final conclusions. Local beliefs attributed disability to: human transgression of social conventions, particularly concerning inappropriate family relations, which invoked a curse; supernatural forces affecting the child; the will of God; unexplained events; and biomedical factors. Challenges associated with disability related to the burden of caregiving and perceived barriers to inclusion, with stress as a shared bi-product. Local understanding of disability in this rural part of Kenya demonstrated overlapping explanations and plurality of beliefs. Two possible interpretations are offered. Firstly, oscillation between explanatory lines demonstrated instability, affecting broader acceptance of disability. Secondly, and more positively, in the face of challenges, the desire to make sense of the existing situation, reflected a healthy pluralism.

## Introduction

Information on the causes of childhood disability is not widely available across communities in low-income countries [1]. Limited support services and poor access to knowledge may be contributing factors [2–3]. Communal narratives have emerged to explain the presence of disability. Underpinned by cultural belief systems [4], such explanations affect, not only the ways individuals with disabilities view themselves, but also the responses of others [5]. Bronfenbrenner’s ecological systems theory defined humans as both a ‘culture-producing species’ and ‘culture produced’ (p.123) [6]. Hence communal narratives, developed in response to the circumstances of disability, may also affect social behaviours and practices. The importance of this dynamic interaction between the person and their environment is now emphasised and enshrined in the World Health Organisation’s biopsychosocial model of disability in the International Classification of Functioning and disability (ICF) [7–8]. In a move away from the earlier medical model, which located the ‘problem’ with the individual, and in recognition of the disabling/enabling role played by the environment, as captured in the social model [9–10], the ICF highlights the bi-directional influences that exist between the person and context. Thus the environment and members of the community have the facility to promote or constrain the abilities, rights and needs of persons living with disability.

### Cultural beliefs

The extent to which culture is a factor in people’s understanding of disability appears to be mediated by the individual’s exposure to people with different disabilities [11–15]. The amount of time and the immediacy of the contact, i.e. in person, were identified as critical factors in the positive attitudes evinced by children [11–14], and physiotherapy students [15]. Allport hypothesised that face to face interactions would have a positive effect on the attitudes of one social group for another [16]. The degree of cognitive maturation is also relevant here [17–19]. Children’s early conceptualisation was found to be consistent with an individual or medical view of disability, characterised by physical and biological factors, and associated with negative and unpleasant experiences [17–19]. Whilst parental understanding of disability was found to be more closely aligned to a social model when given closed response choices, open-ended questions triggered a response based on the most immediate mental representation [17]. Federici et al. [18] suggested that this was evidence of ‘a cognitive mechanism underpinning the cultural construction of the individual/medical model of disability’ (p.7). However, as maturation takes place, cognitive growth appears to open the individual to learning about alternative explanations and views of disability [17–19]. This is where real-life encounters with people who have disabilities and cultural transmission of understanding attain relevance.

Locally-held beliefs about the causes of disability in low-income countries were categorised broadly in an earlier paper by Ingstad [20], as attributable to: “Others”; “Oneself”; and “Fate, nature or the will of God”. Although variations in narratives have been observed [21], some resonate across sub-Saharan Africa. “Others” as an explanatory source may attribute a child’s condition to an external force, such as a curse or evil spirits (‘jinnies’) as reported in Malawi [2], Namibia [22], Tanzania [23], and Kenya [24–26]. “Oneself” implies responsibility for a wrong-doing resulting in disability. For example, angering ancestors by breach of moral code or failing to honour their memory. Improper family relations, including extra-marital affairs and incestuous relationships have been cited as perceived causes of disability with the mothers generally implicated, e.g. in Botswana [27], Ghana [28], Kenya [25–26], Namibia [22], Tanzania [23] and Zimbabwe [29–30]. Ingstad [20] suggested that such stories may be related to social control and the need to adhere to social conventions and moral codes, for example, being chaste in a marriage. However, “Oneself” and “Others’ are not necessarily mutually exclusive. Improper relations attributed to “Oneself”, may trigger a curse affecting the child’s well-being, thereby implicating “Others” as well. For example, it was reported that some Yoruba people believe that atypical developmental conditions are the result of a curse brought about by the defiance of a pregnant woman who walks outside at midday or midnight, or represent a punishment for wrongdoings, such as conducting an extra- marital affair [31]. Sometimes “Oneself” and “Others” combine, as in the causal explanation of albinism in Namibia, where the mother was accused of having had sex with a white man or a ghost [22]. In contrast, Otte et al’s study in Guinea-Bissau, reported quite separate explanations of epilepsy, where over half the respondents with the condition viewed it as caused by evil spirits, with a relative minority viewing it as punishment for a wrongdoing [32].

Ingstad’s last category “Fate, nature, or the will of God” captured the possibility of an unexplained, unplanned circumstance [20]. Theological explanations appear to offer both a positive interpretation (e.g. gift from God) as well as a negative one (e.g. punishment from God). Accordingly, the ascription of different religious meanings has served to justify the challenges of caring for the child [30, 33], implying acceptance and adaptability, or conversely, guilt and insecurity [34]. Such beliefs have been used to rationalise aspects of daily life as part of ‘God’s plan’ [35], with the power of prayer used to address the individual’s problems. Conversely, a more punitive God has been associated with spiritual discontent, with God’s powers connected to punishment [35].

The plurality of explanatory sources used in Eastern Africa has been referred to as “a melting pot of beliefs” (p.394) [36]. For example, respondents at a special school in Tanzania drew on the dominant religions of Christianity and Islam to explain disability, whilst also considering the role of traditional healers [23]. A similar situation was found in Turkana, Kenya where caregivers expressed multiple parallel views [37]. Not restricted to the African continent, the mixing of belief systems was observed in Cambodian parents of children with cerebral palsy who incorporated their Buddhist beliefs into biomedical and traditional explanations [38]. The drive for answers and improvements in the existing situation may trigger changing allegiances to belief systems [23], such that when one explanation and its associated response course fails, there may be a shift to an alternative source of explanation and action.

### Challenges

In the context of a resource-poor country, where the lives of the majority of citizens are characterised by a reduced standard of living and minimal support compared to high-income countries [39], the challenges encountered by people with disabilities are shared by non-disabled individuals [39–40]. This includes poor access to health provision [41], low school attendance [42–43], limited employment rates and low wages [44]. In Africa alone, less than 10% of children with disabilities were reported as attending school [45].

According to Eide and Ingstad the broader conditions of poverty may affect social relations, attitudes, communal beliefs and behaviours towards persons with disability [46]. The way members of a community respond has implications for those closest to the individual, particularly those in caregiver roles. Referred to as ‘courtesy stigma’ [47], and more recently ‘affiliate stigma’ [48], this is the experience of social stigma by association. This compounds the physical, psychological, temporal and financial stresses of the caregiver [24–26, 49–51].

More extreme consequences of stigma and discrimination include neglect and abuse. Sexual abuse has been reported to occur at some time in the lives of 90% of the population with intellectual disabilities [52]. Individuals with communication difficulties were recognised to be at increased risk [53], due to difficulties in reporting abusive incidents to others [54–55]. Adults with disabilities in Malawi disclosed sexual abuse in relation to forced marriages with later abandonment by a marital partner [55]. Family maltreatment of individuals with epilepsy was linked to the perceived disgrace of behaviours associated with convulsive epileptic seizure, such as loss of consciousness, incontinence and myoclonic jerks [32]. However, caregiver actions that are perceived to be negative may be underpinned by more altruistic motives. The Kenya-based CNN documentary, ‘Locked Up and Forgotten’ showed how many children with disabilities were kept apart from the local community in restricted environments, to protect them from the abuses of others and to keep them safe [56]. Whether motivated by negative or positive attitudes, such actions result in the exclusion of people with disabilities from the same places, opportunities and social groupings as other members of the community.

This project was set up to promote disability awareness in neighbourhood communities in a rural part of Kenya. The over-arching aim was to engage people in a process of reflection and education. This paper reports on the first aspect–reflection, which investigated local understanding of disability as a co-constructed concept. The research questions were:

1. What cultural beliefs and knowledge shape local understanding of disability?
2. What challenges are perceived to be associated with disability?

## Materials and methods

To capture the commonly held views and experiences of the community, the research adopted a phenomenological approach with reference to Creswell et al’s description [57].

The setting was Kilifi County, situated on the Indian Ocean coast. The inhabitants were mainly from the Mijikenda groups (about 80%) and spoke Giriama, Chonyi, and Swahili. Christianity was observed by about 70% of the people, traditional religious practices by 20% and Islam by about 10%. Kilifi was among the poorest areas in Kenya, with a poverty level of 71% (Kenya Commission on Revenue Allocation). Most of the rural population live as subsistence farmers, with dwellings of mud construction, and consisting of one or two rooms, no power supply or running water. Per capita, the average income for a household (typically parents plus six children) was Ksh1,000 per month–less than $13 USD [58]. There were low levels of nutrition, inadequate control of infectious diseases, poor enrolment in schools and limited literacy amongst adults generally. Based on a county-wide population of 1,109,735 and using a 15% prevalence of disability [59], it was estimated there were 166,460 people with disabilities in Kilifi County. Therefore, it was envisaged that most people would have experience of people with disabilities in their extended families and communities.

### Ethics

The study was approved by the Scientific Ethics and Review Unit (SERU) in Nairobi Kenya (SSC #1996) and the International Development ethics committee at the University of East Anglia, UK.

### Sample

For expedient recruitment of participants within the timescale, it was decided to focus on established community groups. Locally situated, the groups were considered to be natural and familiar settings for people to come together. The sample was comprised of 21 community groups (263 participants in total) across the five constituencies in Kilifi County. Already constituted, the groups provided a useful entry point to the wider communities. Inclusion of the groups was determined by their: formal organization as community health worker (CHW) groups or Women’s Groups (WG), the former being linked with a health facility [60] and the latter being registered with the Women Microfinance Trust (KWFT) [61]; and their active status with a minimum of once monthly meetings scheduled. The aim was to recruit 4–5 groups per constituency to achieve a cross-section of community groups in the county. A community-based organization called Pambazuko Disability Initiative identified groups who met the selection criteria above, and arranged an initial meeting when project information was disseminated both orally and in paper format. Informed consent was recorded for each group member on a prepared form by participant signature or thumbprint.

There were eleven CHW groups and ten WGs. The CHW groups had a mainly voluntary membership of men and women, ranging from 20–50 years, who came together to assist the government-funded, local health provision. They were recruited by the county public health team and trained in basic health care, although this was reported to be minimal. The WGs were comprised of women only whose membership was voluntary. Their ages ranged from 24–65 years. They were supported by the KWFT, a non-government funded organisation and saved the group’s money in shares, servicing loans to individuals to improve quality of life [61].

### Data collection

Focus groups were carried out with each community group. In order to capture the popular stories in a community, the bringing of people together in a focus group discussion was considered a suitable approach; one that would support review of individual contributions and invite shared recognition of the familiar explanations for disability within the community. The representative from Pambazuko was well known to the community groups. In correspondence with each group’s Chair, she arranged the meetings where the focus group discussions would take place, e.g. at health centres, churches and in classrooms, and arranged the delivery of refreshments. The meetings were opened by the Chair and usually started with a group prayer. The discussion was then introduced by the second author who was a native of the area and proficient in the local languages–Swahili, Giriama and Chonyi. The Pambazuko representative was present at all the meetings.

Participants were encouraged to talk about their experiences of individuals with disabilities residing in their own communities. A discussion guide was followed with each group. Initially, participants were invited to reflect on and share their real-life encounters with people with disabilities. This included living alongside and communicating with such individuals. Then the discussion moved to the causes of disability and problems in communication. Specific mention of ‘communication’ was to support the inclusion of deafness, and its associated impacts, in the groups’ reflections. Finally, the participants were asked what the community could do in response.

The median attendance was 13, with a range of 8–17 participants present at the 21 focus group meetings. Discussions were carried out in the group’s preferred language and recorded on a digital audio device. Using the topic guide, open-ended questions were addressed to the whole group initially and the members were invited to contribute their views and ideas. After the first contributions by members, the questions were re-offered to others in the group in order that conversation floor was shared as equitably as possible. Probes were inserted by the researcher to encourage clarification or topic extension. Once the current discussion appeared to be waning, the next topic would be introduced. On average the discussions took 50 minutes with a few meetings taking over one hour.

The recordings were uploaded to a computer and transcribed by the second author, a native of the area who was conversant in the local languages. The data were transcribed in the local language used in the original discussion, before translation into English. The data were then imported to the data management programme NVivo 10. Throughout the analysis period, checks were carried out on the translation to ensure accurate representation of meanings. This involved an iterative process whereby the first author (native English speaker, UK-based) and the second author reviewed the transcripts, queried emergent concepts and their meanings, using back translation as appropriate.

### Data analysis

Firstly, thematic analysis [62] was used to identify patterns within the data and any interconnections. Initially, the first and second authors analysed the data independently of each other. The transcripts were read in their entirety to establish familiarity with the discourse. Key ideas and concepts were identified and recorded in note form at this stage. This informed the identification of recurrent themes for the creation of a first generation of ‘nodes’ (basic themes) using NVivo-10. The transcripts were reviewed and relevant excerpts were assigned to the ‘nodes’, including a reference to the transcript source. The content of each node was then reviewed and adjustments made to the categorisation as required. Next the ‘themes’ were reviewed for interconnections and grouped under ‘organising’ themes.

The researchers inspected the commonalities and differences in their separate analyses at the levels of basic and organising themes. The second author was able to draw on his background and experience of growing up in this part of Kenya, such that complex local explanations of disability could be unravelled. The other, a UK-based visitor to Kenya for the purposes of disability-focused research, who had accrued some experience in this setting since 2007, contributed a more remote stance, which enabled translations and concepts to be queried and explored in depth. Once consensus on the categories and their labels was achieved at the levels of basic and organising themes, a thematic map was generated. The themes that emerged in relation to research question 1, were resonant of Ingstad’s categories, and it was therefore decided to use Ingstad’s three categories as organising themes, as far as possible (*Onself*, *Others* and *Fate*, *Nature or the will of God*) [20]. Data that were considered unrelated to Ingstad’s categories but still addressed the research question, were grouped according to homogeneity, suitably labelled and added to the thematic map. Lastly, a critical review of possible interconnections at the levels of basic and organising themes was undertaken.

## Results

### Local understanding of disability

**Fig 1:**
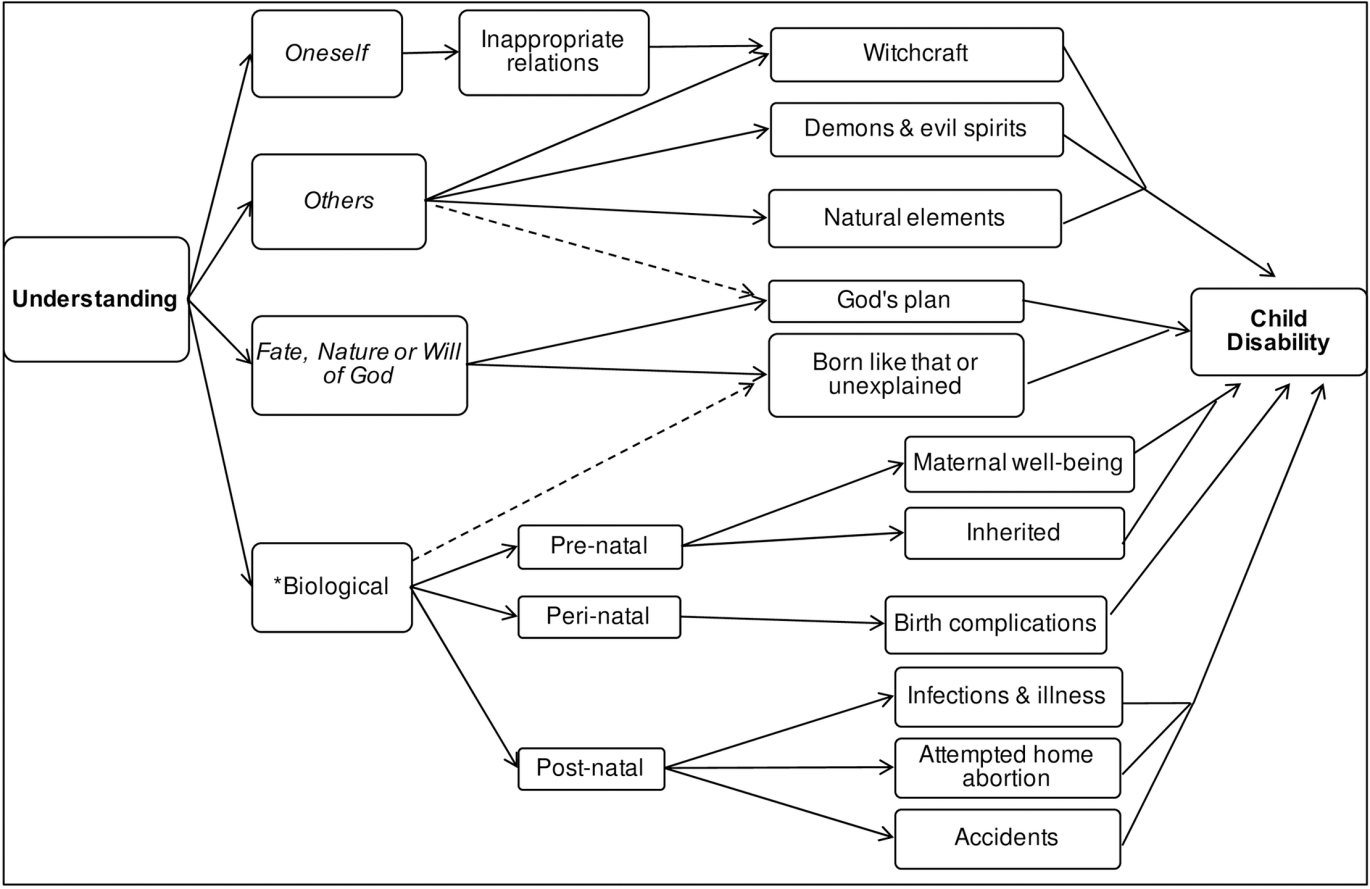
Thematic map summarising local understanding of childhood disability.

Fig 1 illustrates ‘Local Understanding’ through uni-directional arrows connecting the themes, left to right, from organising themes to basic themes relating to ‘Child Disability’. Whilst Ingstad’s categories of cultural belief, represented by italicised titles, were identifiable in the data, they were neither mutually exclusive nor exhaustive. The category *Oneself* expressed as ‘Inappropriate relations’ was said to bring forth a curse issued through ‘Witchcraft’, which was attributable to *Others*. Additionally, some beliefs corresponded to more than one category as indicated by arrows with dashed lines. A fourth category was added to Ingstad’s three original categories, *Biological, which is indicated by an asterisk before the box title.

#### Oneself

More than any other perceived cause of childhood disability, attribution to *Oneself* was supported by the local Giriama culture. The parents, often the mother, were implicated. This was expressed as ‘Inappropriate relations’ or what was referred to as “mixing in the family”, which appeared to be connected closely to family/community values and the flouting of social conventions:

“Disability results from lack of cultural observations; like committing adultery with people in the homestead.” [CHW9]

The perceived improper relations were considered to bring forth a ‘curse’, which affected the perpetrator through the child. Thus belief in an external force responsible for issuing the curse was also implicated, i.e. *Others*. Particular words were reserved for the type of improper relations, although word definitions often seemed to overlap and were used inter-changeably. For example, “mavingane”, “chirwa” and “vitio” were used variously to imply that extra-marital relations had taken place with another family member:

“you will be told that you slept with your younger brother-in-law that is why your child is like this—that is vitio.” [CHW6]

“Mavingane” was also used to refer to adultery. Similarly, “kitio” described improper sexual behaviour in the homestead. In such circumstances, it was common to blame the mother. One respondent explained that the mothers attending the child’s birth would be the first to accuse, upon sight of a newly born child with a disability. The father was said to be implicated ‘second’ or where the innocence of the mother had been proven. However, the word ‘chirwa’ was used to capture the negative effect of the husband’s unfaithfulness during the gestational period. The father’s touch was regarded as tainted with the power to affect the child.

“… when a pregnant mother gives birth to her baby and during that period the husband cheats on the wife and the father comes back and touches the child…” [WG8]

Distinguishing features of disability were described with mention of “humps”, “curved limbs” and “sickness” often associated with different types of transgression. ‘Inappropriate relations’ also extended to sickness in the child and was associated with malnourishment, changes to the skin and general failure to thrive. The gender of the offending parent was sometimes considered relevant also:

“… if it is the father the child crosses the legs and if it is the mother the skin develops wrinkles and stretches his or herself.” [WG9]

“If it is the mother the right leg curves and if it is the father the left one curves.” [CHW6]

The misplacement of household items was also associated with ‘inappropriate relations’. There was mention of (bed) “mats” and “washing” (lines) among the various physical signs purported to bring forth “mavingane”, such as the tying of a rope from the mother’s house to that of the father-in-law for hanging out the washing, and disordered cooking stones. Whether these were believed to be signs that extra-marital relations were taking place, or actual factors in the advent of disability was not clear. It is possible that the narratives contained a cautionary note by recommending against particular actions, which might contravene certain social conventions:

“… you cannot take the mother-in-laws mat and sleep on it or the daughter-in-law gives it to the mother-in-law…” [CHW5]

Interestingly, improper behaviour towards someone with a disability was also perceived as capable of bringing forth a curse that would affect an unborn child. This was recognition of a different type of transgression. For example, laughing at someone because of their appearance was thought to affect the perpetrator’s own offspring eventually. One story told of a woman who laughed at someone with restricted growth and was told by the person that:

“if the baby she was carrying on her back was the last one then he will be tall but if she was still giving birth to more children then she will laugh a lot at home… she gave birth to three dwarfs.” [WG1]

#### Others

As implicated in the narrative of *Oneself* through the issue of a curse, *Others* conveyed the sense of an external force that was in some way responsible for the child’s condition. Fig 1 illustrates how *Others* was expressed through the themes of: ‘Witchcraft’; Demons & evil spirits’; and ‘Natural elements’. The actions of witchcraft were invoked in the circumstance of offence by others. A curse or spell was cast as retribution for a past transgression, with disability viewed as the negative consequence.

“… other people can put a spell on the Mother or in that family, so when the parents give birth they give birth to helpless children… the child instead gives trouble to the parents.” [CHW2]

However, this did not exclude the possibility of other causes. For example, it was recognised that certain types of disability may be attributable to sickness or unexplained reasons, whilst others to a preternatural power:

“There is disability … that one is born with and there is another that one gets through sickness and there is another through witchcraft…” [CHW7]

‘Demons & evil spirits’ dominated group views of disability causation. The cause of disability was explained by an external force. The person with a disability was sometimes described as having been placed under or on a seat for demons or ghosts. Explanations not only defined the external, supernatural force in disability causation, but also identified concomitant physical symptoms and financial gains. Mention of drooling (dripping saliva) by the individual with a disability was sometimes related to the perceived wealth of a parent, often the father, or another person. Thus disability in one person appeared to be explained by suspect financial gain in a relative.

‘… the work that the child does is just to sit there while the saliva is dripping; he is just in a terrible condition while the father there has a lot of money.’ [CHW5]

The phrase “borrowing the pregnancy” was used to describe how the parents would have visited a shrine to help them have a baby. In this circumstance, references were made to mythical creatures and evil spirits:

“…others who are also born … cannot be understood if they are human or animal-like … because the pregnancy was forced. They went and borrowed it from the ogres.” [CHW10]

Less frequent reference was made to the ‘Natural elements’, specifically the influence of the moon and sea, which was used principally to rationalise the occurrence of epileptic seizures.

#### Fate, nature or will of God

The *Will of God* as per Ingstad’s categorisation [15], interpreted disability as part of ‘God’s plan’. However, it could also be said to be attributable to *Others;* although a more philosophical stance was implied, which was associated with acceptance and recognition of fate or nature. This contrasted with the more negative themes in the *Others* category:

“I think he was born like that because the father and the mother are fine. So I think God created him like that.” [WG2]

Such beliefs appeared to satisfy not only an explanation for and acceptance of the child’s condition, but also provided a source of hope that things would change for the better. Stories were told of miracles whereby a disability was somehow cured:

“…he was disabled and the mother looked for help from everywhere she gave up and left it in the hands of the Lord. She stayed one day only, the next day he was walking by himself and everybody was amazed.” [WG4]

Relating to *Fate* and Nature, there was recognition that some babies were “born like that”, which included the “unexplained”. It appeared there was an overlap with the fourth category, *Biological, which was added to Ingstad’s three. Acceptance of the child’s condition or the notion that some events were beyond human control, appeared to be implicit in this interpretation. This represented a direct counterpoint to cultural beliefs focusing on *Oneself* and *Others*.

“You can get out of your mother’s womb as a disabled child.” [WG4].

#### Biological

As well as accounting for the ‘unexplained’, Biological attribution defined tangible events or biomedical circumstances that were likely responsible for an individual’s condition. It was expressed in three key areas as illustrated in Fig 1: ‘Pre-natal’; ‘Peri-natal’; ‘Post-natal’.

‘Inherited’ conditions’ were mentioned sparingly, where a family history or a grandparent with a similar problem was proffered as an explanation for occurrence in a later generation. Maternal well-being issues regarding antenatal care were more frequently mentioned, but maternal age was implicated in a single reference to the menopause. Certain conditions or actions were observed to be counter to a safe pregnancy and implicated *Oneself*, such as the mother being infected by a sexually transmitted disease or taking drugs, massaging the stomach or sexual relations with the husband during confinement.

“… the parents did not relax concerning matters of the bedroom that is why the child became blind.” [CHW4]

The mother’s health and nutritional level were recognised as important to the baby’s well-being, alongside the role of medical services in maintaining the mother’s health during pregnancy. A lack of attention to antenatal care was viewed as a possible reason for a child to be born disabled.

“… there are those of us who never visit the hospital throughout the entire period of pregnancy you might find that the child maybe born disabled.” [WG1]

Family planning, particularly the use of contraceptive pills, was viewed with suspicion by some respondents and considered responsible for a child born with:

“… no senses’ or ‘… without eyes” [WG8]

Stories were told of “attempted home abortion” using some form of medicine that was not successful and instead affected the baby:

“… she wanted to abort the baby … used drugs but by bad luck the pregnancy did not come out, but when she gave birth to the child the time had not yet arrived and the child was terrible because one of the hands was clawed and the leg was a half.” [CHW6]

‘Birth complications’ focused on difficulties in breathing during or after birth. There was also mention of twisting of strangulation by the umbilical cord, placental difficulties causing bleeds, and ingestion of liquid variously affecting the unborn child. Birth struggles and the need for help during delivery were also cited causes.

Post-natally, a range of ‘infections and illnesses’ was identified including malaria, polio, yellow fever and febrile convulsions. Inadequate vaccination was seen as the cause, with delayed or lack of treatment a possible factor in any resulting disability:

“… some of the children I have seen, were infected with malaria, and when they get treatment it is okay, but when they miss treatment quickly, they get disabled.” [WG9]

Epilepsy was said to have developed as a later consequence of some forms of illness. The respondents described the development of sores and boils on the child’s body and the disabling consequences that were observed. Stroke as a source of disability was mentioned in relation to blood pressure problems in adults. ‘Accidents’, mainly transport related, were viewed solely as affecting adults, and referred to the loss of a limb and altered motor function as possible consequences. Of concern was the negative effect on the person’s ability to work. However, no mention was made of other impacts that may not be immediately visible, such as acquired brain injury.

#### Plurality of beliefs

Far from being completely remote to each other, the cultural beliefs shaping understanding of disability appeared to be interconnected. The preference for one narrative was not to the exclusion of another. Rather it was common for two or more explanatory lines to run concurrently, and even be applied together to a given situation in the pursuit of change for the better, if not a cure for the child’s disability:

“the child has been taken to hospitals and the child has also to the church for prayers but the parents were told that she was attacked by demons and till now the child is just the same.” [CHW5]

The quest for a treatment to improve the child’s condition was a clear driving force. Occasionally, change for the better was attributed to one particular course of action that aligned itself to one of the narrative genres:

“I tried everything possible. In fact he had amulets on the hand and leg. But the doctors really helped a lot.” [WG10]

### Challenges

**Fig 2:**
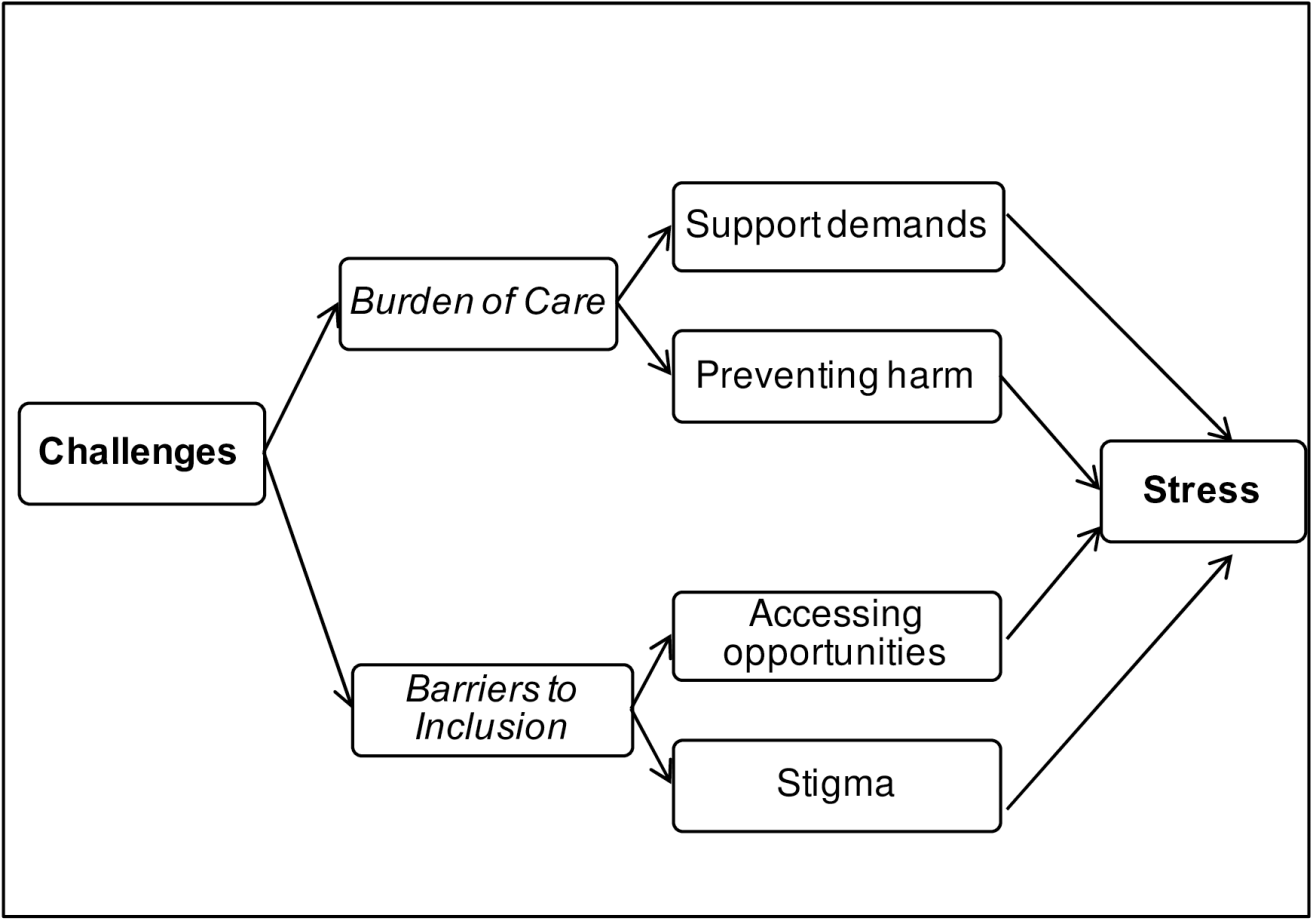
Thematic map summarising the perceived challenges associated with disability.

Challenges were recognised in relation to disability, both for the person affected, and their family, particularly the caregiver. As illustrated in Fig 2, these were organised around two organising themes: *Burden of Care* and *Barriers to Inclusion*. *Caregiver stress*, which included psycho-social, physical and financial factors, emerged as a third theme with connections to both the other two.

### Burden of care

Associated with disability, this was related to ‘support demands’ and ‘preventing harm’. Challenges concerned personal maintenance of the individual and family in terms of clothing, hygiene, mobility and sustenance. This extended to self-care, and caring for your children when you have a disability yourself. Stories of ‘support demands’ told of individuals who were unable to swallow food placed in their mouth, who would discard their clothes once dressed, had poor motor control and problems with continence, and who were dependent on others for bathing.

“she has to be bathed and removed from where she has been washed and she is carried by two people and put back to the bed because she is not able to move on her own.” [WG7]

Beyond everyday care demands, management of the individual’s behaviour was a common concern. This included behaviour that impacted on others in routine events. For example, “grabbing of food” away from others:

“if you don’t take away the food and he comes and he sees other children eating he will just grab the food.” [WG2]

Alongside ‘support demands’, there was recognition of the potential for exploitation and harm of the person who was unable to decipher dangerous situations. The need to manage the environment and reduce the degree of risk to the child’s health and wellbeing resonated across the discussions.

“When the parent leaves the house she has to remove everything and hide it because it is a must for him/her to take it, for example if it is kerosene he will take it and even drink it and do with it other messy things.” [CHW2]

Sometimes the groups spoke of actions to either conceal the presence of the person, or else to protect them from harm. For example, one respondent told of a boy tied up to prevent him from roaming and forcing himself on a woman.

#### Barriers to inclusion

Whilst the *Burden of Care* featured in many of the stories told by the respondents, insights into the *Barriers to Inclusion* were also shared. Difficulties in ‘accessing opportunities’ were recognised with individual experiences, such as going to school, being constrained. There was a sense that many children and young people with disabilities stayed within the confines of the homestead and were not part of the surrounding community. Empathy was expressed for known individuals as the challenges of accomplishing routine functions and transactions were described. Communication difficulties were attributed to many people familiar to the groups, which included problems with self-expression and understanding the communications of others. The perceived problems of individuals who were deaf were talked about particularly.

“When she is asked she cannot talk, she is pregnant and she has not finished school……” [CHW1]

Concern was expressed about the mobility and comfort of some individuals with motor problems, with a lack of suitable equipment, such as wheelchairs, and the physical pain endured by some also. Even when a wheelchair was available, the challenges of navigating unmade roads and a limited transport system were evident:

“they gave him a wheelchair but even coming to buy something from the shop was a problem because the road was terrible.” [CHW1]

Alongside ‘accessing opportunities’ there was ‘stigma’. The child was viewed as being tainted in some way and therefore not deserving of the same recognition as a non-disabled child. Reference was made to a child being physically rejected by the family, as in excluding them from the home or being “thrown away” [CHW2].

“when you want to mention something about the disabled you will find that the Mother does not want to speak of it…. it’s like she has already changed the topic.” [WG8]

The desire to conceal the child was expressed in two main ways. Firstly, there was denial of disability in conversation, whereby a mother was described as having “already changed the topic” [WG8]. Secondly, and demonstrating a more extreme response to the negative view of disability, there was talk of children being restrained in some way with chains or ropes. Interactions with the child were described as showing a lack of human warmth and regard, thereby assigning the child to a sub-human class:

“She locks the child up in a house and when she is being given food she pushes the food to her using a stick. The child looks as if he/she has not taken a bath for ages.” [CHW2]

The groups told of many incidents of unfair and abusive treatment of the person with a disability. Stories were told of children being chased away if they asked for food and of individuals being “falsely judged”. When something happened that was undesirable or caused a problem, it was often the case that the individual with a disability would be blamed and punished:

“He sleeps at home with the other children and they wet the bed but he is the only one who will be beaten and will also wash the clothes of the other children.” [CHW5]

The stigma associated with people with disabilities extended to people trying to help. The implication was that the person offering assistance would also “…give birth to such a child” [CHW7].

#### Stress

Stress appeared to be a bi-product of both ‘Burden of care’ and ‘Barriers to inclusion’, affecting not only the caregiver, but also the individual with a disability. Unable to leave their child for any period of time due to on-going care needs, caregivers were described as having “trouble”. Often this depicted the tensions between making sure the child was looked after whilst also needing to go to work in order to contribute to the household income:

“the mother is experiencing a lot of trouble because when she wants to go somewhere … she cannot carry the child and cannot leave her at home also.” [CHW4]

The “trouble” experienced by individuals with disabling conditions was also articulated, either as a general recognition of the difficulties experienced in everyday life or more specifically in relation to parenthood and employment:

“She has problems, she has a child with no job and the times are hard. So these are things which are sad.” [CHW1]

## Discussion

Ingstad’s closely connected categories [20] of *Oneself* and *Others* reflected Giriama philosophy in this part of the Kenyan coast, but were also resonant of narratives in other sub-Saharan countries including Malawi [2], Namibia [22], Tanzania [23] and Zimbabwe [29]. Generally, disability was associated with negative images, which is consistent with the representations used by children [17–19], and in the automatic responses of adults [17]. An underlying cognitive process may be at work here, as suggested by Federici et al. [18], such that when open-ended questions were posed to the groups, there was immediate resort to an early mental representation. Attributing the child’s condition to some form of malevolent, preternatural force, by reference to demons, evil spirits and witchcraft, contributed to the view of disability as both undesirable and unacceptable.

Lacking exposure to alternative explanatory models, which might have supported a different set of interpretations [17], the language used and its associated visualisations shifted the view from what Goffman described as ‘a whole person to a tainted, discounted one’ (p.3) [47]. However, attributing disability to the flouting of moral codes, such as indulging in extra-marital relations and incest between members of the same family, at least deflected the focus from the individual with a disability to others [20]. To the extent that answers were sought in the immediate environment, this would seem to be reminiscent of a social model [9–10]. However, causal blame was still inferred, albeit attributed to immediate family members, and the predominant view of the child was as a flawed being. Women were particularly identified as complicit, although not exclusively so, in traditional stories of immoral behaviour, possibly commensurate with their generally lower status in African society and their roIes as caregivers and managers of the homestead. In this way disability was located in a social context, which is consistent with the idea of sense-making [36]. Explanations for disability were further supported by references made to the physical world. For example, the tying of a washing line was connected to incestuous relations in the family; curvature of spine or limbs represented the effects of a curse; and saliva production was linked to demons and ill-gotten financial gain. Thus the intangible, i.e. the preternatural forces, assumed a real world form in the communal stories with references made to misplacement of household objects and physical manifestations of a child’s disability.

The data showed that religious beliefs provided a possible remedy for a major life stressor such as disability, which is also corroborated in the literature [33–34]. References to the ‘will of God’ demonstrated cognitive appraisal of circumstances and rationalisation of responses [23, 33–34]. The faith-based explanations revealed a benevolent presence, with no mention of the punitive God reported by others [27, 35]. Thus religion was seen as a coping strategy, which entailed benign reappraisal of faith and the seeking of religious support. As Pargament suggested, this appeared to be related to acceptance and adjustment [34]. This is different from the published findings of others [22, 31–32] where a punitive God was also recognised.

*Biological* attributions of disabling conditions told of biomedical causes related to pre-, peri- and post-natal difficulties. The participation of CHW groups, who were attached to a local health dispensary, may account for the growth in knowledge. Engaged in health matters and interacting with trained practitioners on a voluntary basis may account for their awareness of biomedical matters. However, the WGs also cited biological causes of disability. Some of the women belonged to both types of community group and thus cross-fertilisation of information was likely. It is worth noting that whilst some explanations appeared to have a scientific basis, myths with no substantive foundation were also reported, e.g. taking birth control pills. Thus the quality of biomedical information about the causes of disability was observed to be emerging, but inconsistencies in understanding were present. Never-the-less, it was evident that the views expressed by the groups were, in part, attributable to a learning process [17].

In the circumstance of sparse information [1] and poor access to limited resources [2–3], competing explanations appear to have developed–some based on biomedical evidence, and some on superstitious beliefs. Thus a cultural repertoire of disability meanings and causes existed. It is possible that pragmatism lies at the centre of these plural belief systems (*Others; Oneself; Fate*, *nature or the will of God; and Biological*). Explanatory narratives were closely associated with the pursuit for answers to the disability question and the quest to change the given situation for the better [27]. For example, *Biological* attribution conveyed a health problem where a visit to a medical centre might follow. In contrast, *Others* or *Oneself* implied some form of wrongdoing or the presence of an evil force might necessitate a visit to a local witchdoctor. Failure to rectify the situation through one course of action seemed to trigger a shift from one belief system to another, and to a different course of action. It appeared that the major sources of explanation for disability were not mutually exclusive; rather they operated as components of a spectrum defined by the desire to understand and improve the given situation.

The communal narratives were set against a backdrop of perceived challenges of caring for someone with a disability, or indeed living with a disability. The difficulties associated with caregiving and meeting ‘support demands’ in the circumstance of limited resources were recognised factors. This is consistent with earlier research evidence, with much of the burden of caregiving falling to the mother [24–26, 49–51]. The respondents were not only aware of the stresses placed on caregivers, but also recognised the difficulties experienced by some individuals with disabilities. The sharing of stories and the sympathy expressed regarding the challenges faced, indicated that many of the groups’ members had encountered people with disabilities and their caregivers in their local communities, which may have tempered their individual perspectives [11–16]. Efforts made to protect the child from physical hazards in the homestead, as well as from the aversive responses of others, formed another dimension of the caregiver burden. The desire to ‘prevent(ing) harm’ reflected the narratives presented in the Kenya-based CNN documentary, ‘Locked Up and Forgotten’ [56]. Of course, the very act of keeping children with disabilities apart from the community, may contribute to the social distance between them.

Constraints on community inclusion were defined by challenges of accessing opportunities and the existence of stigmatising attitudes. Practices that excluded the individual from the places, activities and events shared by other members of the community were consistent with the findings of others [40–45], and undermined possibilities and opportunities for those with disability [4], particularly in terms of education and livelihood [34–45].

In a context of poverty, limited resources and psychological stresses, people with disabilities appeared to be assigned a lower priority. Groce observed that cultural beliefs about disability causation may have attracted prejudicial attitudes and discriminating behaviour within the family unit, and beyond in the community [21]. However, an alternative explanation for exclusionary behaviours was also evident in the desire of some caregivers to protect their child from personal harm and to prevent anti-social behaviour. Thus, in addition to the plural belief systems used to explain disability, the practices observed in relation to people with disabilities may also have had more than one type of rationalisation.

### Limitations

Focus group discussions were conducted in formally constituted community groups. Whilst this brought the benefits of a familiar membership in a natural setting, serving to authenticate the popular explanations for disability, the purpose of the groups, the health focus of CHW groups and the address of female interests in the WGs, were also potential sources of influence. A purposive sample of members of the local community may have yielded a more representative data set. The membership of the CHW groups was mixed compared to the female membership of the WGs. In terms of discursive process, however, no gender-specific issues were observed. High participant numbers in some of the FGDs may have constrained the flow of discussion, although the alternative of capping the number invited into the discussion was considered a risk to community engagement. The qualitative study focused solely on FGDs and therefore triangulation of data was not carried out. As the first part of a two-phase study, the focus was on reflection. Feedback to the groups was restricted to the intervention phase (education) where disability awareness training was carried out. This is reported separately.

## Conclusions

In a context of poverty, limited information, poor education and access to resources, a repertoire of communal narratives has emerged to support local understanding of disability. The current study, whilst supporting the relevance of Ingstad’s categories of cultural beliefs [20] in this part of Kenya, defined the inter-connections between the different categories. Biomedical factors were also present within the framework of cultural understanding. Plurality of beliefs was common, and closely linked to challenges associated with the burden of caregiving and barriers to inclusion. In the face of such difficulties, it appeared that efforts were made to make sense of the existing situation. It could be said that oscillation between explanatory lines demonstrated instability, where the search for understanding and cure affected broader acceptance of and adaptability to the circumstance of disability. Alternatively, a more positive interpretation asserts that the repertoire of explanatory narratives reflected a healthy pluralism, whereby multiple and sometimes combined courses of action were part of efforts to make a difficult and challenging situation better. Further research is needed to examine the relationship between cultural beliefs and local responses, and the motivations underpinning particular courses of action.

## Supporting information

S1 File Focus group discussion guide. (DOCX)
