# Supplementary material for: The perception of disability by community groups: Stories of local understanding, beliefs and challenges in a rural part of Kenya
Source: PLoS One. 2017 Aug 3;12(8):e0182214. doi: 10.1371/journal.pone.0182214 (PMC5542666; doi:10.1371/journal.pone.0182214)
Supplement: S1 File — (DOCX) [file pone.0182214.s001.docx]

**Focus Group Discussion Guide:**

**English version:**

1: Have you had an encounter with a person with a disability? What kind of a person was s/he? What were the difficulties? What could s/he do?

2: Have you had communication problems with a person who has a disability? What were the difficulties? What could s/he do?

3: What do you think are the causes of disability and communication difficulties?

4: What can the community do about these disabilities and difficulties experienced by such individuals?

**Giriama version:**

1: Udzangwe kuona hedu kushi na mutu mwenye ulemavu? Were ahizho? Shidaze were ni noni? Were anadima kuhenda noni?

2: Udzangwe kukala na shida ya kuwasiliana na mutu mwenye ulemavu? Shidaze were ni noni? Were anadima kuhenda noni?

3: Unafikiri ulemavu na shida za kuwasiliana zinarehewa ni noni?

4: Lalo rinadima kuhenda noni dzulu za ulemavu na shida za kuwasiliana ambazo aa atu manazipata?

**Kiswahili version:**

1: Ushapata kuona au kuishi na m,tu mwenye ulemavu? Alikuwaje? Shida zake zilikuwa nini? Alikuwa anaweza kufanya nini?

2: Umepata shida ya kuwasiliana na mtu mwenye ulemavu? Shida zake zilikuwa nini? Alikuwa anaweza kufanya nini?

3: Unafikiria ulemavu na shida za kuwasiliana zinasababishwa na nini?

4: Jamii inaweza kufanya nini juu ya ulemavu na shida za mawasiliano ambazo watu hawa huwakumba?
